# Subglacial lake drainage detected beneath the Greenland ice sheet

**DOI:** 10.1038/ncomms9408

**Published:** 2015-10-09

**Authors:** Steven Palmer, Malcolm McMillan, Mathieu Morlighem

**Affiliations:** 1 College of Life and Environmental Sciences, University of Exeter, Exeter EX4 4RJ, UK; 2 Centre for Polar Observation and Modelling, School of Earth and Environment, The University of Leeds, Leeds LS2 9JT, UK; 3 Department of Earth System Science, University of California Irvine, Irvine, CA 92697, USA

## Abstract

The contribution of the Greenland ice sheet to sea-level rise has accelerated in recent decades. Subglacial lake drainage events can induce an ice sheet dynamic response—a process that has been observed in Antarctica, but not yet in Greenland, where the presence of subglacial lakes has only recently been discovered. Here we investigate the water flow paths from a subglacial lake, which drained beneath the Greenland ice sheet in 2011. Our observations suggest that the lake was fed by surface meltwater flowing down a nearby moulin, and that the draining water reached the ice margin via a subglacial tunnel. Interferometric synthetic aperture radar-derived measurements of ice surface motion acquired in 1995 suggest that a similar event may have occurred 16 years earlier, and we propose that, as the climate warms, increasing volumes of surface meltwater routed to the bed will cause such events to become more common in the future.

## Abstract (web summary)

How the Greenland ice sheet responds to the drainage of water below the ice remains uncertain. Here, the authors combine digital elevation models and Landsat optical imagery to map subglacial drainage pathways and investigate the ice sheet surface response to a periodically draining subglacial lake.

## Graphical abstract

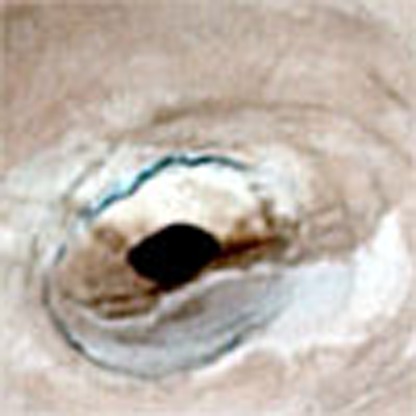

The Greenland ice sheet (GrIS) has lost mass to the global oceans at an increasing rate in recent decades^1^,^2^. Surface meltwater runoff accounts for roughly half of the GrIS ice loss^3^,^4^; yet the pathways that transport water to the ice margin remain poorly understood^5^. High rates of water flow through an inefficient subglacial system have the capacity to drive temporary accelerations in ice velocity, as observed when supraglacial lakes drain^6^. Similarly, outburst floods from subglacial lakes can induce an ice dynamic response, a process that has been observed in Antarctica^7^, but not yet in Greenland, where the presence of subglacial lakes has only recently been discovered^8^,^9^.

Meltwater is generated at the surface of the GrIS during summer and flows under gravity in supraglacial streams, collecting in local topographic lows and forming supraglacial lakes. The locations of these topographic lows reflect the underlying subglacial topography^10^ and so these are not advected as the ice flows toward the margin. As melting continues through the summer months, these supraglacial lakes and streams can connect to englacial and subglacial pathways through moulins and crevasses. While it is typically assumed that water at the base of the ice sheet flows unimpeded to the ice margin, subglacial water residence times and drainage network efficiency remain important unknowns that affect the response of ice dynamics to increased meltwater drainage^5^.

Remote sensing observations have shown increased ice melt extent and intensity in recent years^11^,^12^,^13^,^14^, with greater increases in melt extent for the Western GrIS than for the Eastern GrIS^11^,^15^,^16^. Although some previous studies have predicted enhanced sensitivity of ice sheet motion to increased melting, retreat and thinning^17^, the processes linking meltwater production to ice dynamics are not well understood, and the response of the ice sheet to future warming remains uncertain^5^. For instance, it has been hypothesized that periods of enhanced sliding during warmer-than-average summers are followed by winters, during which the ice sheet flows slower than average, due to the time taken for an efficient channelized subglacial system to return to a distributed system typical of winter^18^,^19^. Results from modelling studies suggest that short-term variability in meltwater input is key to driving enhanced ice sliding^20^, and several studies have observed temporary speed-ups in response to increased melting or discrete meltwater pulses from supraglacial lake drainages^6^,^21^,^22^,^23^,^24^,^25^,^26^.

In this study, we infer for the first time the subglacial drainage pathway from a subglacial lake in Greenland, and the dynamics of this subglacial channel. It is clear that the mechanism of dynamic response of the ice sheet to meltwater input is sensitive to the nature, and evolution, of the subglacial hydrological system.

## Results

### Ice surface collapse basin

**Figure 1:**
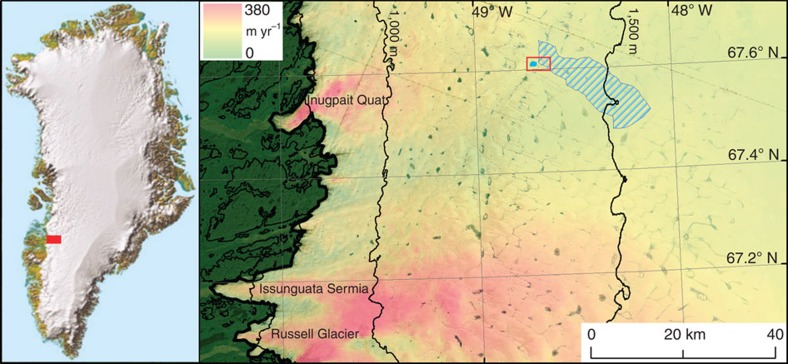
Context of study area. Outline of collapse basin (blue polygon), and supraglacial catchment (blue hatching) shown in relation to the western margin on the Greenland ice sheet. Wintertime ice flow^44^ and contours of ice sheet surface elevation^28^ at 500 m intervals are also shown. The red rectangle indicates the area shown in Fig. 2.

**Figure 2:**
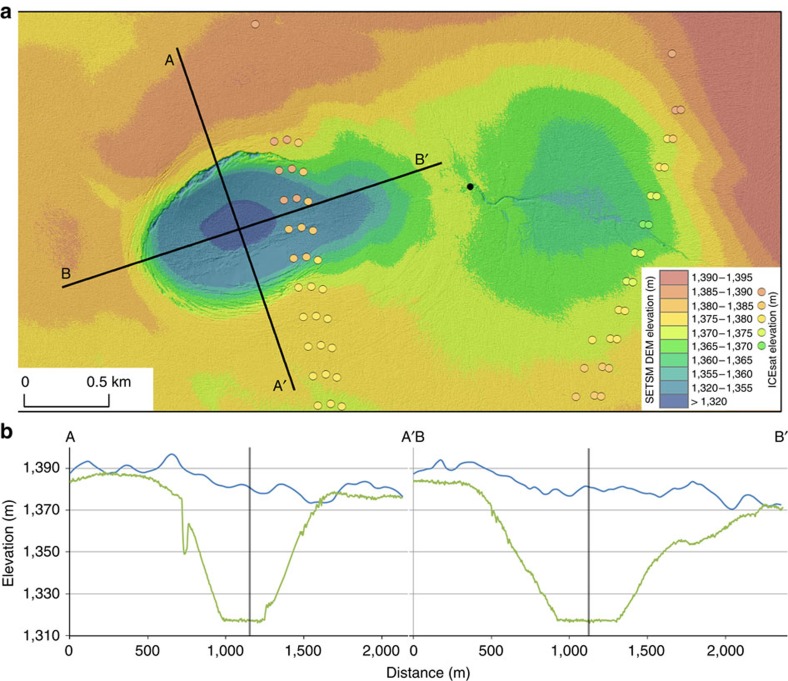
Topography of collapse basin. (**a**) Surface elevation of the collapse basin and nearby supraglacial lake basin (in metres above WGS84 ellipsoid) from SETSM (colour bar), and ICESat (coloured circles), overlaid on a hillshaded version of the SETSM DEM. The location of the nearby moulin is shown as a black circle. (**b**) Profiles of surface elevation in 2007 from the GIMP DEM (blue) and 28 October 2011 from the SETSM DEM (green). The vertical bars show where the two profiles intersect.

**Figure 3:**
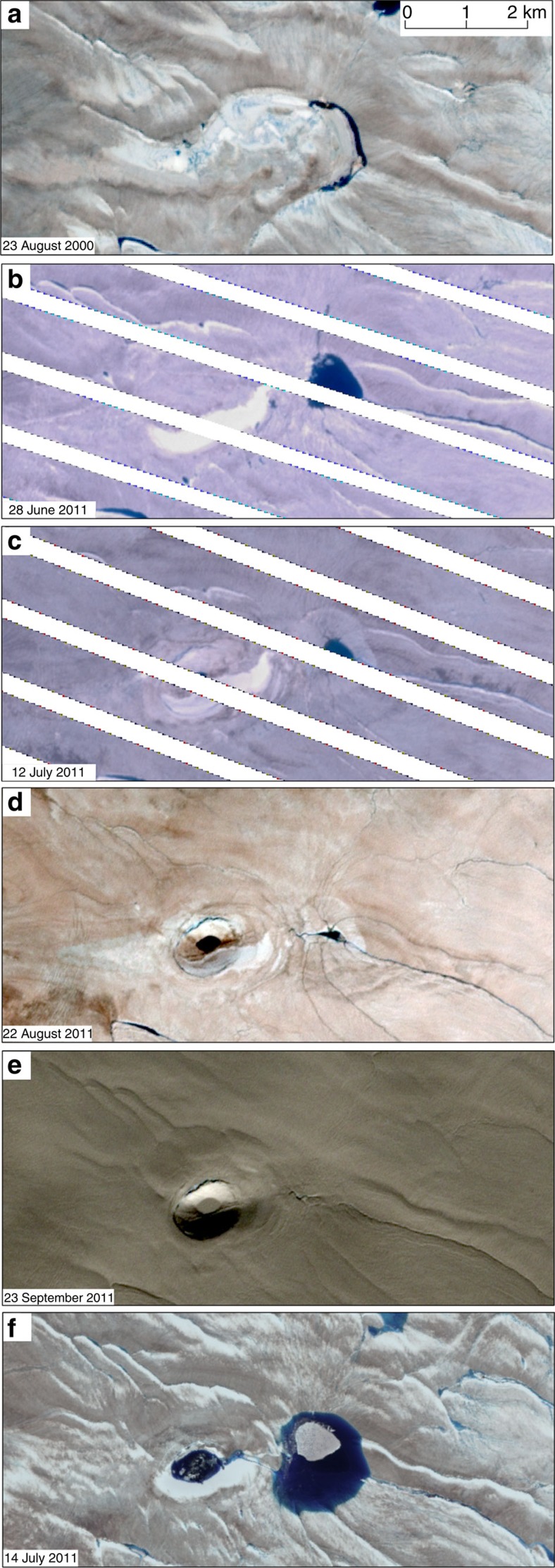
Timing of collapse basin formation. Sequence of Landsat 7 (**a**–**e**) and Landsat 8 (**f**) optical imagery showing the formation of the collapse basin between 28 June 2011 and 12 July 2011 and its persistence to the present day. The missing lines in panels **b** and **c** are due to the scan line corrector malfunction on Landsat 7. Imagery provided by the USGS.

We used surface extraction with triangulated irregular network-based search-space minimization (SETSM)^27^ digital elevation models (DEMs) derived from satellite imagery acquired on 28 October 2011 to reveal the presence of a 62±4 m deep collapse basin in the ice sheet surface, situated 48 km inland from the land-terminating Inugpait Quat glacier at the western margin of the GrIS (48.70W, 67.61N; Fig. 1). This feature is oval in shape, measuring 1.6 by 1.1 km and covering an area of 1.4 km^2^. No evidence of the feature is detected in the Ice Cloud and Land Elevation Satellite (ICESat) data acquired in 2005 or in the ice elevation data from the 2007 Greenland Ice Mapping Project^28^ (Fig. 2). In order to negate the effects of ice surface elevation changes over the 4-year period caused by factors such as variations in surface mass balance or large-scale ice flow, we estimated the downward displacement of the collapse basin by comparing the elevation offset between the feature rim and its centre as measured by the 2007 and 2011 data sets (Fig. 2). We observe that this elevation offset increased from 3±4 m in 2007 to 62±4 in 2011, that is, the vertical displacement over the 4-year period was 59±6 m, with an average downwards displacement over the whole basin of 34±6 m. A sequence of Landsat optical imagery shows that the collapse basin formed during a 2-week period between 28 June 2011 and 12 July 2011, and that it persists to the present day (Fig. 3).

### Mechanism of formation

**Figure 4:**
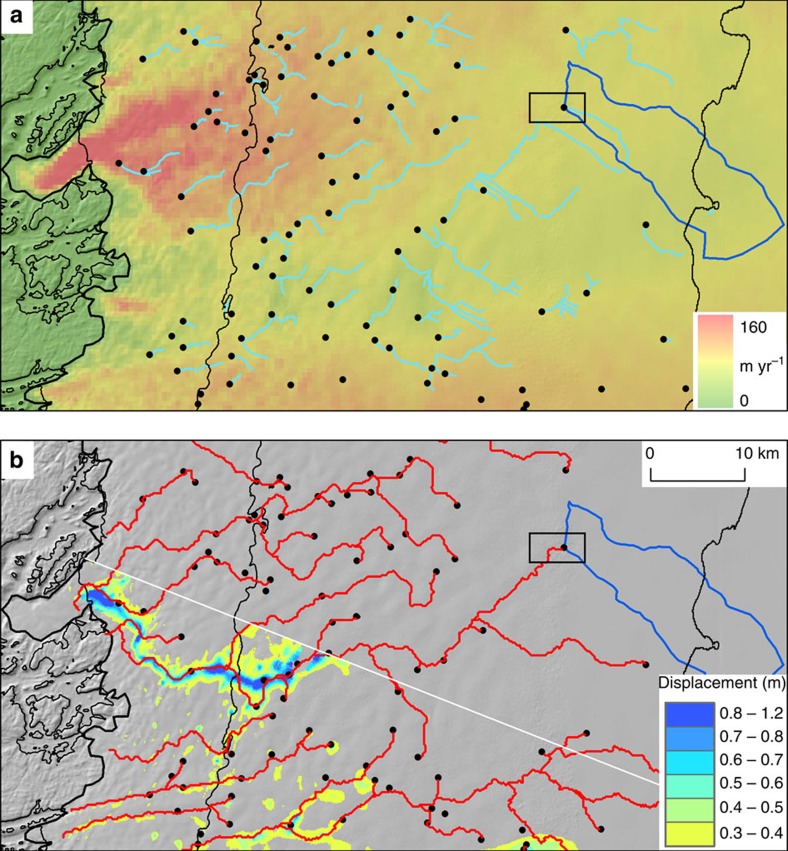
Ice surface and bed hydrology and motion at the western margin of the GrIS. (**a**) Distribution of observed supraglacial streams (light blue) and inferred moulins (black dots) over winter flow speed^44^ and (**b**) modelled subglacial water pathways (red) and InSAR-derived downward displacement in metres (yellow to blue). The background is a hillshaded version of the SETSM DEM. The collapse basin shown in Fig. 2 is highlighted by the black box. The supraglacial catchment drained by the moulin in Fig. 1 is shown outlined in dark blue. The thick black line indicates the ice sheet margin, the thin black line indicates 500 m elevation contours and the white line is the northern extent of the InSAR data.

To investigate this unusual feature, we mapped supraglacial streams and moulins in the vicinity of the collapse basin using the SETSM DEMs (Fig. 4). The collapse basin is located near a supraglacial lake, which partially drained between 28 June and 12 July 2011 (Fig. 3) along an ∼500 m long supraglacial stream into a moulin located <1 km to the East of the collapse basin (Figs 2 and 4a). To investigate where water transported through this moulin to the bed would be routed, we modelled the subglacial drainage pathways, and found that the collapse basin is located directly above a major subglacial drainage pathway (Fig. 4b). Concentric tension fractures visible around the perimeter of the feature in Fig. 2 (up to 35 m deep and 40 m wide) are consistent with a rapid localized downwards movement of the ice sheet. Given this evidence, and the similarity of this feature to those observed in Antarctica where subglacial lakes have drained^29^,^30^,^31^, we interpret the collapse basin to have been formed following the rapid drainage of a subglacial lake with a volume of 4.8±0.8 × 10^7^ m^3^.

The flat and horizontal floor of the collapse basin shown in the SETSM data acquired in October 2011 (Fig. 2) suggests that the central ice was in hydrostatic equilibrium at that time, that is, some water remained in the lake basin. This means that our calculated subglacial lake volume is likely to be an underestimate. An explanation for this remaining water is that the subglacial lake had started to refill with supraglacial meltwater drained via the nearby moulin during the 4 months, since it drained in late-June/early-July 2011.

## Discussion

Unlike Antarctica, where subglacial lakes are fed by meltwater generated at the ice sheet base, the proximity of the inferred subglacial lake to a moulin suggests that the lake may have been storing surface-derived meltwater. We therefore compared our estimate of the water volume discharged from the subglacial lake, which we assumed to be equivalent to the ice volume of the collapse basin, to that stored in the nearest supraglacial lake. Using the SETSM DEMs and Landsat imagery, we estimated that this supraglacial lake had a maximum area of 0.66±0.075 km^2^, an average depth of 3±1 m, and held a maximum volume of 2.0±0.9 × 10^6^ m of water. This equates to roughly 1/24 of the water volume required to form the collapse basin and suggests that the volume of water evacuated from the bed could not be supplied solely by the nearest supraglacial lake, even over many annual cycles of supraglacial lake filling and draining (Fig. 2a).

Considering lakes within the wider vicinity, we estimated that the supraglacial catchment feeding the moulin via supraglacial streams drains 180 km^2^ of the ice sheet^32^, and contains seven supraglacial lakes with a combined estimated volume of 4.0 (±1.4) × 10^7^ m^3^. While this is within the uncertainty of the estimated volume of the inferred subglacial lake, it is important to note that not all lakes in the catchment drain every summer, so it would take more than a single melt season to accumulate enough supraglacial meltwater to fill the subglacial lake. Our analysis demonstrates the potential for this feature to accumulate supraglacial meltwater from a wide area, and over several summers, via drainage of supraglacial water through a nearby moulin. Therefore, we suggest it is able to discharge large pulses of water into the subglacial system in a more punctuated way than the draining of nearby supraglacial lakes individually.

Although a lack of ice-penetrating radar observations over the collapse basin prevents us from identifying why the lake formed in this location, we speculate that it is located immediately upstream of topographic ridge, too small to be resolved by the ice thickness data set. The existence of such a ridge would form a hydropotential ‘dam', resulting in an area of lower hydropotential immediately upstream, which could store any free water at the bed. If thick sediments underlay the ice sheet, as suggested by recent seismic observations^33^, it is likely that this ‘dam' is at least partially made of deformable sediment. We suggest that once the subglacial lake had collected sufficient surface meltwater to overtop this barrier, the lake drained rapidly through a subglacial tunnel until empty, whereby erosion caused by the escaping water led to a positive feedback in which water discharge and dam erosion are mutually reinforcing. For temperate ice, this mechanism of subglacial water transport through tunnels is well established^34^. We estimate a minimum mean discharge rate over the 14-day period (as indicated by the Landsat data in Fig. 4) to be 30 m^3^ s^−1^, which is similar to that calculated for an Antarctic subglacial lake drainage event^35^. It is possible that the drainage event occurred over a much shorter period than this, in which case the discharge rate would be correspondingly higher.

The rapid formation and partial refilling of the collapse basin suggests a large, and temporally limited, input of water into the downstream subglacial system (Fig. 3b), with the capacity to significantly alter its hydrological characteristics episodically. To investigate the fate of the water drained subglacially from the lake, we used the interferometric synthetic aperture radar (InSAR)-derived ice sheet surface motion data shown in Fig. 4. The data show localized lowering and/or acceleration of the ice sheet surface along a 30 km section of the modelled drainage pathway of the subglacial lake (Fig. 3b), with a line-of-sight (LOS) displacement of up to 1.3 m along the narrow channel of ice (300–1,500 m wide, average ∼1 km) occurring between 11 and 12 August 1995. We suggest that this unusual pattern of surface displacement above the subglacial flow route was caused by the ice-overburden closure of a subglacial R-channel, following the drainage in early August 1995 of the subglacial lake located below the collapse basin observed in 2011. While we cannot eliminate the possibility that the large volume of drained subglacial water required to form the linear slump feature originated from the other branch of the subglacial drainage pathway not covered by the 1995 InSAR data (Fig. 4b), the lack of any evidence since 1995 to indicate the presence of a subglacial lake near this other branch makes this less likely than our suggestion that it originated from the observed subglacial lake.

Schoof^20^ modelled subglacial conduit formation and closure in response to meltwater flow and found that water input variability was the primary driver of short-term glacier velocity increases. The subglacial lake drainage we detect represents a mechanism by which the short-term amplitude of this variability in water inputs may be increased substantially, but is a process that has received little attention to date. Observations of discrete melt inputs from supraglacial lake drainages show ice speed-ups lasting for only ∼1 day, suggesting that the subglacial environment can drain large volumes of water relatively efficiently^36^. However, our results suggest that here the volume of water stored subglacially is an order of magnitude larger than the volume stored in a typical supraglacial lake, so it is expected to have a larger and longer-lived impact on ice flow speeds in the short term. If melt extent and intensity in Greenland increases as expected, we suggest that similar subglacial lake drainage events will occur with increasing frequency, resulting in a higher degree of channelization at the bed forming earlier in the melt season. This, in contrast to the expected short-term response, may limit the effect of enhanced sliding at the bed due to increased melting^37^.

We have revealed the presence of a 1.4 km^2^ collapse basin on the surface of the GrIS, which we interpret as being the surface expression of a drained subglacial lake. The estimated drained water volume is larger than that can be stored within a nearby supraglacial lake, and must have been delivered from a larger supraglacial catchment accumulated over several melt seasons. While the first two subglacial lakes to be detected in Greenland have been observed recently at the periphery of the ice sheet^8^, this is the first inference of a subglacial storage and release of water in the main body of the ice sheet. Furthermore, these results provide the first indication that in Greenland, like Antarctica, subglacial lake drainage may occur beneath the ice sheet and can modify the hydrological system. Comparison of these recent observations with InSAR observations made 16 years earlier suggests that this subglacial lake has drained in the past and is likely to drain episodically. If the recent trend of increasing melt extent and intensity continues^11^,^12^,^13^,^14^, it is likely that subglacial lake drainage will become more common, as any subglacial lakes will overtop their hydropotential ‘dams' more frequently. While this episodic storage and release of water subglacially will change the basal thermal regime by delivering additional heat to the subglacial system, the more extensive development of efficient subglacial drainage channels earlier in the melt season may limit the effect of increased melting on enhanced sliding.

## Methods

### SETSM DEMs

SETSM^27^ DEMs are gridded surface elevation data constructed from overlapping pairs of high-resolution (∼0.5 m) images acquired by the DigitalGlobe, Inc. Worldview 1 and 2 satellites through the National Geospatial-Intelligence Agency (NGA) enhanced view license. The DEMs are built using photogrammetric techniques in which common features are identified in each image and are used to model the relative three-dimensional position of the terrain. They are constructed without ground control and rely on the satellite-positioning model to locate the surface in space. The accuracy of each DEM is primarily limited by the accuracy of the rational polynomial coefficients used in the positioning model and are estimated to have a vertical accuracy of ±3 m (ref. 9). Supraglacial hydrological features such as meltwater streams are clearly visible in these SETSM DEMs, and were manually digitized using standard GIS software.

### ICESat elevation data

To provide additional information about ice surface elevation, we used satellite laser altimeter measurements acquired by the GLAS instrument^38^ on board NASA's ICESat. During 2005, three repeats of a reference track crossing the lake site were made, providing surface elevation profiles before the formation of the collapse basin. The sensor has a ∼50 by 60 m elliptical footprint and samples elevation every ∼170 m along the satellite track with a single shot accuracy of the order of 10 cm (ref. 39). We used Release 633 of the GLA06 product, applying the sensor saturation range correction supplied and removing measurements where it was lacking.

### Landsat imagery

To aid mapping the temporal evolution of features on the ice surface, we produced true colour composites using Level 1B true colour Landsat 7 (2000–2011) and Landsat 8 (2014) downloaded from the US Geological Survey (USGS) Earth Explorer website. For each image, the panchromatic band 8 was used to increase the image resolution from 30 to 15 m. Some of the Landsat 7 images used suffer from missing data due to the well-known scan line corrector failure.

### InSAR ice surface motion measurements

We processed two pairs of synthetic aperture radar (SAR) images acquired during August 1995 to derive ice sheet motion^4^ interferograms over two 24-h periods. These data were acquired during the European Remote Sensing Satellite tandem phase, a unique data SAR operational period that has not been repeated or duplicated since, meaning that similar, more recent observations are not possible. The interferometric baseline of the main SAR image pair acquired on 11/12 August 1995 was 0.5 m, meaning that the phase contribution due to topography is negligible^40^. Interferograms detect motion in the LOS of the radar instrument, which, due to the side-looking geometry, contains both a horizontal and a vertical component. While it is not possible to determine the relative contributions from the two components for an individual interferogram, it is typical in InSAR studies of ice motion to assume that all ice flow vectors are parallel to the ice surface^5^. However, altimetry and field-based studies^6^,^7^ have shown that vertical motion of the ice surface, associated with movement of water at the ice sheet bed, can occur. We attempt to isolate this vertical component of ice motion by differencing the early August interferogram with a topographically corrected late-August interferogram (30/31 Aug 1995), under the assumption that the large-scale horizontal flow of the ice sheet would not have changed significantly in the 19-day interval between acquisition of the SAR image pairs. Any residual motion detected in the radar LOS is assumed therefore to be due primarily to changes in vertical motion of the ice sheet surface occurring between the two 24-h observation periods, though any anomalous changes in horizontal flow will also be recorded. We note that as the European Remote Sensing Satellite SAR antenna look angle is inclined 23° from the vertical, it is ∼2.4 times more sensitive to the vertical motion of the ice sheet surface than to horizontal motion.

### Hydrological modelling

To estimate where subglacial water would be routed at the base of the ice sheet, we assumed that water would flow in the direction of the steepest hydropotential gradient, and that the effective pressure is zero, that is, that the water pressure at the bed is equal to the weight of overlying ice^41^. Basal hydropotential was calculated using ∼400 m resolution ice thickness and bed elevation data gridded at 150 m, derived by applying a mass conservation algorithm to NASA's operation ice bridge ice thickness measurements^42^. We used hydrological modelling routines^43^ within a standard desktop GIS software package to predict where free water would be routed assuming an impermeable subice surface, with all local topographic minima ‘filled' and water flow in the direction of the steepest hydropotential slope. Moulins were inferred to exist where supraglacial streams identified from the SETSM DEMs terminated abruptly, and these were used as the locations of water input for the subglacial hydrological flow routing.

## Additional information

**How to cite this article:** Palmer, S. *et al*. Subglacial lake drainage detected beneath the Greenland ice sheet. *Nat. Commun.* 6:8408 doi: 10.1038/ncomms9408 (2015).
